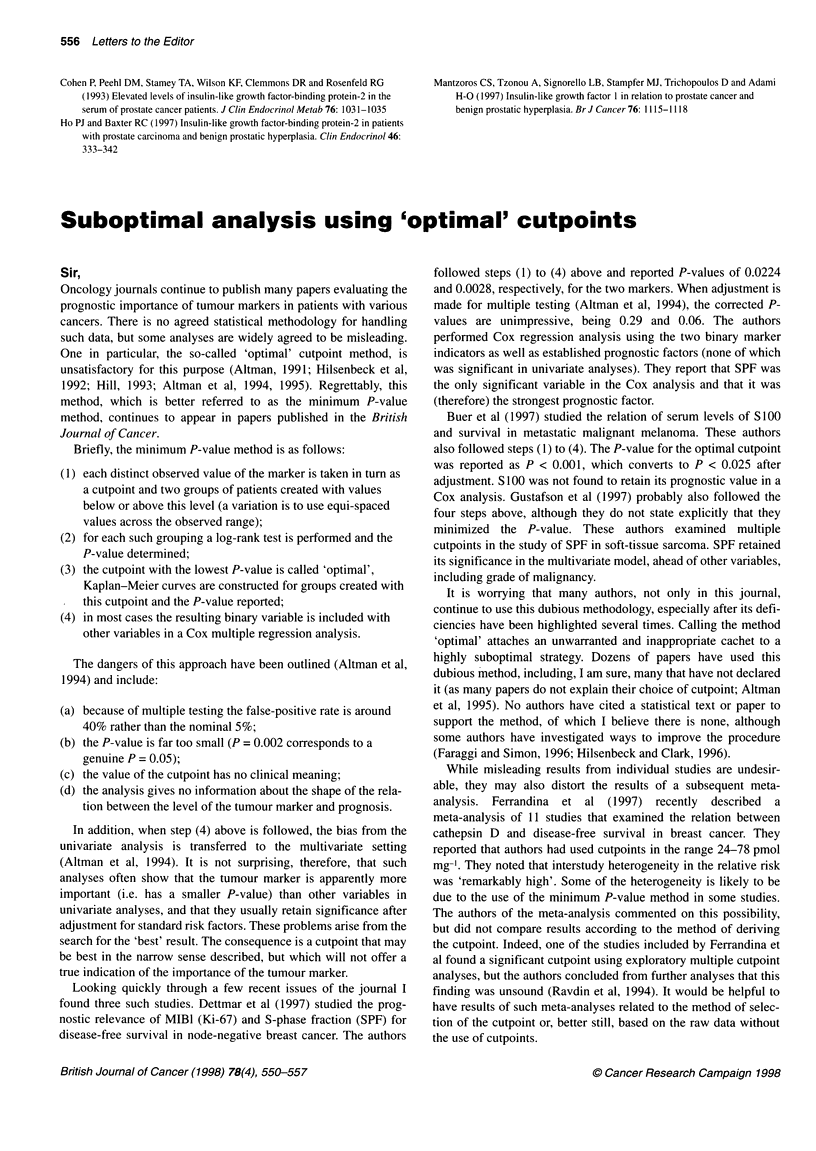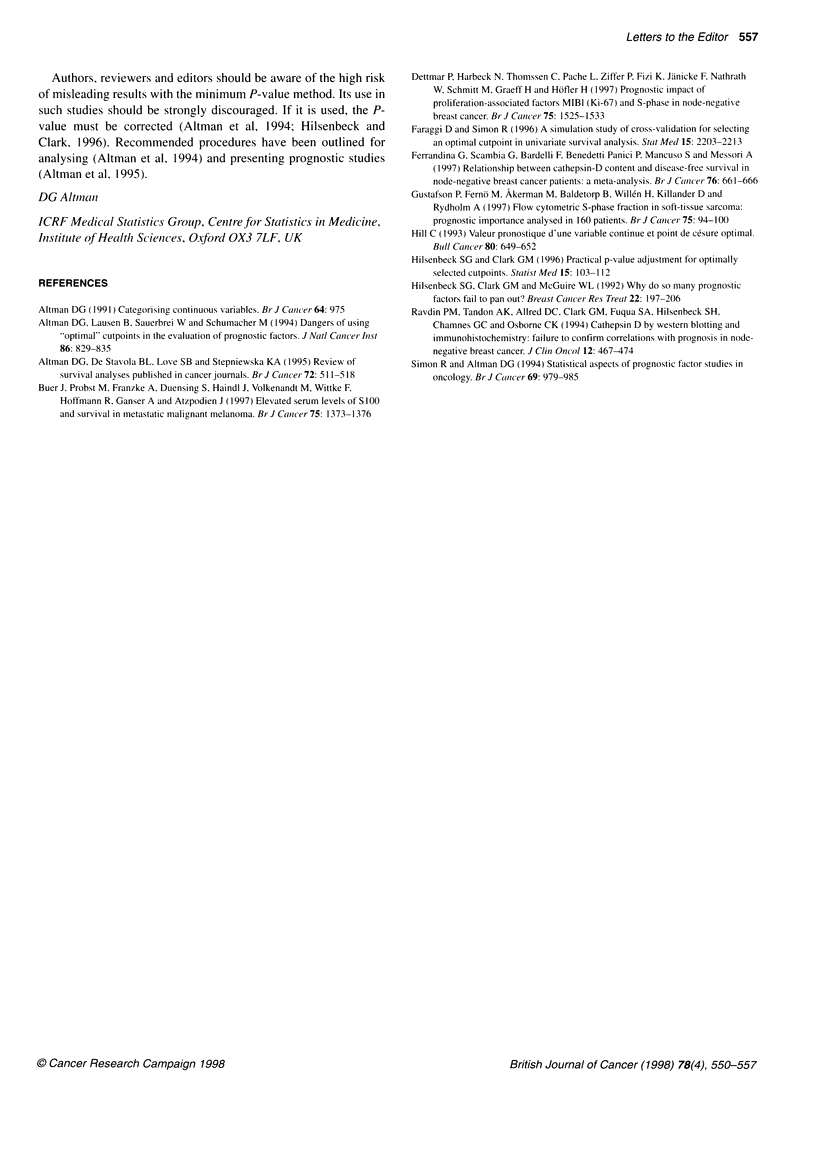# Suboptimal analysis using 'optimal' cutpoints.

**DOI:** 10.1038/bjc.1998.537

**Published:** 1998-08

**Authors:** D. G. Altman


### Suboptimal analysis using 'optimal' cutpoints

**Sir,**

Oncology journals continue to publish many papers evaluating the prognostic importance of tumour markers in patients with various cancers. There is no agreed statistical methodology for handling such data, but some analyses are widely agreed to be misleading. One in particular, the so-called 'optimal' cutpoint method, is unsatisfactory for this purpose (Altman, 1991; Hilsenbeck et al, 1992; Hill, 1993; Altman et al, 1994, 1995). Regrettably, this method, which is better referred to as the minimum *P*-value method, continues to appear in papers published in the *British Journal of Cancer*.

Briefly, the minimum *P*-value method is as follows:

(1) each distinct observed value of the marker is taken in turn as a cutpoint and two groups of patients created with values below or above this level (a variation is to use equi-spaced values across the observed range);
(2) for each such grouping a log-rank test is performed and the *P*-value determined;
(3) the cutpoint with the lowest *P*-value is called 'optimal', Kaplan–Meier curves are constructed for groups created with this cutpoint and the *P*-value reported;
(4) in most cases the resulting binary variable is included with other variables in a Cox multiple regression analysis.

The dangers of this approach have been outlined (Altman et al, 1994) and include:

(a) because of multiple testing the false-positive rate is around 40% rather than the nominal 5%;
(b) the *P*-value is far too small ($P = 0.002$ corresponds to a genuine $P = 0.05$);
(c) the value of the cutpoint has no clinical meaning;
(d) the analysis gives no information about the shape of the relation between the level of the tumour marker and prognosis.

In addition, when step (4) above is followed, the bias from the univariate analysis is transferred to the multivariate setting (Altman et al, 1994). It is not surprising, therefore, that such analyses often show that the tumour marker is apparently more important (i.e. has a smaller *P*-value) than other variables in univariate analyses, and that they usually retain significance after adjustment for standard risk factors. These problems arise from the search for the 'best' result. The consequence is a cutpoint that may be best in the narrow sense described, but which will not offer a true indication of the importance of the tumour marker.

Looking quickly through a few recent issues of the journal I found three such studies. Dettmar et al (1997) studied the prognostic relevance of MIBl (Ki-67) and S-phase fraction (SPF) for disease-free survival in node-negative breast cancer. The authors followed steps (1) to (4) above and reported *P*-values of 0.0224 and 0.0028, respectively, for the two markers. When adjustment is made for multiple testing (Altman et al, 1994), the corrected *P*-values are unimpressive, being 0.29 and 0.06. The authors performed Cox regression analysis using the two binary marker indicators as well as established prognostic factors (none of which was significant in univariate analyses). They report that SPF was the only significant variable in the Cox analysis and that it was (therefore) the strongest prognostic factor.

Buer et al (1997) studied the relation of serum levels of S100 and survival in metastatic malignant melanoma. These authors also followed steps (1) to (4). The *P*-value for the optimal cutpoint was reported as $P < 0.001$, which converts to $P < 0.025$ after adjustment. S100 was not found to retain its prognostic value in a Cox analysis. Gustafson et al (1997) probably also followed the four steps above, although they do not state explicitly that they minimized the *P*-value. These authors examined multiple cutpoints in the study of SPF in soft-tissue sarcoma. SPF retained its significance in the multivariate model, ahead of other variables, including grade of malignancy.

It is worrying that many authors, not only in this journal, continue to use this dubious methodology, especially after its deficiencies have been highlighted several times. Calling the method 'optimal' attaches an unwarranted and inappropriate cachet to a highly suboptimal strategy. Dozens of papers have used this dubious method, including, I am sure, many that have not declared it (as many papers do not explain their choice of cutpoint; Altman et al, 1995). No authors have cited a statistical text or paper to support the method, of which I believe there is none, although some authors have investigated ways to improve the procedure (Faraggi and Simon, 1996; Hilsenbeck and Clark, 1996).

While misleading results from individual studies are undesirable, they may also distort the results of a subsequent meta-analysis. Ferrandina et al (1997) recently described a meta-analysis of 11 studies that examined the relation between cathepsin D and disease-free survival in breast cancer. They reported that authors had used cutpoints in the range 24–78 pmol $mg^{-1}$. They noted that interstudy heterogeneity in the relative risk was 'remarkably high'. Some of the heterogeneity is likely to be due to the use of the minimum *P*-value method in some studies. The authors of the meta-analysis commented on this possibility, but did not compare results according to the method of deriving the cutpoint. Indeed, one of the studies included by Ferrandina et al found a significant cutpoint using exploratory multiple cutpoint analyses, but the authors concluded from further analyses that this finding was unsound (Ravdin et al, 1994). It would be helpful to have results of such meta-analyses related to the method of selection of the cutpoint or, better still, based on the raw data without the use of cutpoints.

© Cancer Research Campaign 1998

Authors, reviewers and editors should be aware of the high risk of misleading results with the minimum *P*-value method. Its use in such studies should be strongly discouraged. If it is used, the *P*-value must be corrected (Altman et al, 1994; Hilsenbeck and Clark, 1996). Recommended procedures have been outlined for analysing (Altman et al, 1994) and presenting prognostic studies (Altman et al, 1995).

*DG Altman*

*ICRF Medical Statistics Group, Centre for Statistics in Medicine, Institute of Health Sciences, Oxford OX3 7LF, UK*

### REFERENCES

Altman DG (1991) Categorising continuous variables. *Br J Cancer* **64**: 975

Altman DG, Lausen B, Sauerbrei W and Schumacher M (1994) Dangers of using "optimal" cutpoints in the evaluation of prognostic factors. *J Natl Cancer Inst* **86**: 829–835

Altman DG, De Stavola BL, Love SB and Stepniewska KA (1995) Review of survival analyses published in cancer journals. *Br J Cancer* **72**: 511–518

Buer J, Probst M, Franzke A, Duensing S, Haindl J, Volkenandt M, Wittke F, Hoffmann R, Ganser A and Atzpodien J (1997) Elevated serum levels of S100 and survival in metastatic malignant melanoma. *Br J Cancer* **75**: 1373–1376

Dettmar P, Harbeck N, Thomssen C, Pache L, Ziffer P, Fizi K, Jänicke F, Nathrath W, Schmitt M, Graeff H and Höfler H (1997) Prognostic impact of proliferation-associated factors MIBl (Ki-67) and S-phase in node-negative breast cancer. *Br J Cancer* **75**: 1525–1533

Faraggi D and Simon R (1996) A simulation study of cross-validation for selecting an optimal cutpoint in univariate survival analysis. *Stat Med* **15**: 2203–2213

Ferrandina G, Scambia G, Bardelli F, Benedetti Panici P, Mancuso S and Messori A (1997) Relationship between cathepsin-D content and disease-free survival in node-negative breast cancer patients: a meta-analysis. *Br J Cancer* **76**: 661–666

Gustafson P, Fernö M, Åkerman M, Baldetorp B, Willén H, Killander D and Rydholm A (1997) Flow cytometric S-phase fraction in soft-tissue sarcoma: prognostic importance analysed in 160 patients. *Br J Cancer* **75**: 94–100

Hill C (1993) Valeur pronostique d'une variable continue et point de césure optimal. *Bull Cancer* **80**: 649–652

Hilsenbeck SG and Clark GM (1996) Practical p-value adjustment for optimally selected cutpoints. *Statist Med* **15**: 103–112

Hilsenbeck SG, Clark GM and McGuire WL (1992) Why do so many prognostic factors fail to pan out? *Breast Cancer Res Treat* **22**: 197–206

Ravdin PM, Tandon AK, Allred DC, Clark GM, Fuqua SA, Hilsenbeck SH, Chamnes GC and Osborne CK (1994) Cathepsin D by western blotting and immunohistochemistry: failure to confirm correlations with prognosis in node-negative breast cancer. *J Clin Oncol* **12**: 467–474

Simon R and Altman DG (1994) Statistical aspects of prognostic factor studies in oncology. *Br J Cancer* **69**: 979–985

© Cancer Research Campaign 1998